# Gut dysbiosis in primary sarcopenia: potential mechanisms and implications for novel microbiome-based therapeutic strategies

**DOI:** 10.3389/fmicb.2025.1526764

**Published:** 2025-01-28

**Authors:** Wei Yang, Si-Cong Si, Wei-Hua Wang, Jing Li, Yi-Xin Ma, Huan Zhao, Jia Liu

**Affiliations:** Department of Geriatrics, Xuanwu Hospital, Capital Medical University, National Clinical Research Center for Geriatric Diseases, Beijing, China

**Keywords:** sarcopenia, primary sarcopenia, muscle mass, gut microbiota, probiotic, short-chain fatty acids

## Abstract

Primary sarcopenia is characterized by a progressive loss of skeletal muscle mass, strength, and physical function that occurs with aging. Despite the related adverse or even serious health outcomes, no medications are currently available for treating primary sarcopenia. Here, we discuss recent advancements in understanding the mechanistic role of gut microbiota-muscle cross-talk in primary sarcopenia, and the therapeutic implications. The mechanistic insights encompass a causal role of gut dysbiosis in primary sarcopenia, potentially mediated through gut microbiota-derived bioactive metabolites, such as short-chain fatty acids (SCFAs), secondary bile acids, and their associated signaling pathways, which may be translated into the development of new microbiome-based treatment and diagnostic approaches. Furthermore, we identify challenges that need addressing in future studies to facilitate the translation into potential novel treatment and differential diagnosis for older individuals with sarcopenia.

## Introduction

Sarcopenia, a medical term initially introduced in 1989, is a skeletal muscle disorder, with primary sarcopenia defined as an age-related progressive loss of skeletal muscle mass, strength, and physical function (Cruz-Jentoft and Sayer, 2019; Petermann-Rocha et al., 2022). This condition is notably prevalent, ranging from 10 to 27% in individuals aged 60 years and older, as reported by a recent systematic review and meta-analysis (Petermann-Rocha et al., 2022). Amid global population aging, primary sarcopenia remains a significant health challenge worldwide. In older individuals, sarcopenia is commonly linked to substantial adverse outcomes, including an increased likelihood of falls and fractures, functional impairments, a decreased quality of life, and a higher risk of mortality (Cruz-Jentoft and Sayer, 2019). Furthermore, sarcopenia can cause disability, resulting in a loss of independence, which, along with other adverse outcomes, may require long-term care placement or caregiver assistance, thereby imposing a substantial social and economic burden. At present, it is known that primary sarcopenia is multifactorial and not limited to age-related lifestyle changes (e.g., physical inactivity and low-protein diet), inflammation, and insulin resistance (Houston et al., 2008; Paddon-Jones et al., 2008; Liu and Zhu, 2023; Mo et al., 2023; Sánchez-Sánchez et al., 2024; Xie and Wu, 2024). These factors contribute to alterations in muscle protein turnover as well as the development and progression of sarcopenia. However, the extent of other factors that may contribute to sarcopenia development is not fully understood, and the precise underlying mechanisms of sarcopenia remain elusive. Currently, no medications are available for the treatment of this skeletal muscle disorder, but nonpharmacological interventions (e.g., exercise regimens and nutritional supplementation) have been recommended to manage sarcopenia (Lo et al., 2020). Deciphering the underlying mechanisms and guiding the development of effective preventive and treatment strategies are considered priorities in sarcopenia research.

The gut microbiota, consisting of over 100 trillion bacterial cells, plays a vital role in human metabolic and immunological health (Agus et al., 2021; Mancin et al., 2023). The gut microbiota can produce a wide range of bioactive compounds, mainly including short-chain fatty acids (SCFAs), secondary bile acids, branched-chain amino acids, and many others, to impact host physiology and health via different host-microbe metabolic pathways (Mancin et al., 2023). Gut microbes break down indigestible carbohydrates, primarily dietary fibers and resistant starches, and to a lesser extent, proteins, through fermentation to produce SCFAs (butyrate, acetate, and propionate) (Ross et al., 2024; Wilson et al., 2020). SCFAs have demonstrated multiple functions in the gut and beyond after entering the bloodstream. Studies have shown that SCFAs serve as an energy source for the epithelium and regulate colonic mucosal inflammation and proliferation in the gut (Ross et al., 2024). Once in the bloodstream, they exhibit epigenetic and immunomodulatory effects on various organs, contributing to the development of a range of human diseases, including primary sarcopenia (Ross et al., 2024; Fusco et al., 2023).

Gut dysbiosis, an imbalanced gut microbiota resulting from compositional changes, has been associated with aging (Coman and Vodnar, 2020; DeJong et al., 2020) as well as various age-related health conditions and diseases, including sarcopenia (Hawley, 2020). Gut dysbiosis and sarcopenia commonly occur in older individuals. Convincing evidence from animal and human studies has linked gut dysbiosis to sarcopenia, with a recent study suggesting a causal relationship (Liu et al., 2021; Zhao et al., 2023). Particularly, the recent Mendelian randomization analysis, a technique used to determine causal relationships between a risk factor and an outcome, a causal association was unveiled between gut microbiota and sarcopenia-related traits, such as appendicular lean mass and low hand-grip strength (HGS) (Zhao et al., 2023). Substantial progress also has been achieved in understanding the effects of gut dysbiosis and the related changes in microbiota-derived metabolites on skeletal muscle metabolism (Mancin et al., 2023). For example, bile acids, initially synthesized in the liver and subsequently metabolized by the intestinal microbiota, play a critical role in modulating host metabolic pathways through activating nuclear receptors like the farnesoid X receptor (FXR) (Mancin et al., 2023). Gut dysbiosis can potentially cause skeletal muscle atrophy through the bile acid–FXR signaling pathway (Mancin et al., 2023). The newly acquired knowledge in understanding the connections between the gut microbiota, main metabolites, and skeletal muscle mass may reveal potential targets that may be translated into the development of new microbiome-based treatment and diagnostic approaches.

While the factors influencing human gut microbiomes are complex throughout the lifespan, age itself has been shown to adversely affect the diversity of gut microbiomes and their beneficial metabolites (Bradley et al., 2024). This impact may contribute to age-related diseases, including sarcopenia. Studies have demonstrated that *Bifidobacterium* and *Lactobacillus* supplements enhance muscle mass and strength in aged mice (Ni et al., 2019), and similar benefits have been observed with a prebiotic formulation in elderly individuals (Buigues et al., 2016). However, it remains unclear whether there is a direct impact of gut microbiota on muscle mass, function, and the development of sarcopenia. It is also challenging to pinpoint the specific gut microbiomes and their metabolites that are beneficial to muscle health and could serve as therapeutic targets. Additionally, it is still elusive how the gut microbiome and its metabolites regulate the gut-muscle axis, which is indeed a topic of ongoing investigation. Further research is needed to fully understand the mechanisms and to explore potential therapeutic interventions targeting the gut microbiota to prevent or treat sarcopenia, thus promoting healthy aging.

As such, we conducted this review by accessing the PubMed, Google Scholar, and Med Online databases as of September 30, 2024 to discuss the recent advancements in understanding the mechanistic role of gut microbiota-muscle cross-talk in the pathogenesis of primary sarcopenia as well as the therapeutic and diagnostic implications. Additionally, we identify challenges and propose future directions to facilitate the translation into potential microbiome-based interventions and differential diagnosis for older individuals with sarcopenia, ultimately enhancing care for sarcopenia in the elderly population.

### Prevalence of primary sarcopenia and its associated health issues

Sarcopenia poses a growing global health concern among the elderly, including in China. Currently, there is a lack of uniform standard diagnostic criteria for sarcopenia worldwide. Various international groups, including the Asia Working Group for Sarcopenia (AWGS), the International Working Group on Sarcopenia (IWGS), the European Working Group on Sarcopenia in Older People (EWGSOP), and the Foundation for the National Institutes of Health (FNIH) Sarcopenia Project, have each issued their diagnostic criteria and definitions for sarcopenia (Fielding et al., 2011; Chen et al., 2014; Studenski et al., 2014; Chen et al., 2020). The variations in diagnostic criteria and definitions may contribute to the differing prevalence estimates of sarcopenia across the globe. For instance, in a longitudinal multicenter cohort study, sarcopenia was defined using various diagnostic criteria established by different international groups (Liu et al., 2020). The prevalence of sarcopenia among community-dwelling older adults (aged 50 years and older) varied from 3.3 to 17.5% according to the different diagnostic criteria applied, with FNIH at 3.3%, AWGS at 9.1%, IWGS at 16.1%, and EWGSOP at 17.5% (Liu et al., 2020). In a more recent study, Petermann-Rocha et al. (2022) conducted a systematic review and meta-analysis to determine the global prevalence of sarcopenia in those aged 60 years and above and found that it varied from 10 to 27%, with the lowest rate in Oceania and the highest rate in Europe using the EWGSOP criteria.

Several previous studies have examined the prevalence of sarcopenia in older Chinese adults from community-dwelling populations, nursing homes, and clinical settings (Tian et al., 2017; Wu and Li, 2019; Chen et al., 2021). Chen et al. conducted a meta-analysis of 58 observational studies involving a large sample of individuals aged 60 years and older in China, residing in community settings, nursing homes, and hospitals (Chen et al., 2021). This study utilized the AWGS diagnostic criteria to assess the prevalence of sarcopenia, and the results indicated higher prevalence rates among elderly individuals in nursing homes (26.3% for men, 33.7% for women), hospitals (29.7% for men, 23.0% for women), and community-dwelling older adults (12.9% for men, 11.2% for women) (Chen et al., 2021). These prevalence rates among community-dwelling older adults in Chen’s study align with the results of two earlier studies involving a sample of community-dwelling elderly individuals and using the AWGS criteria, which reported prevalence rates of 11% for males and 10% for females (Wu and Li, 2019), and 14% for males and 9.11% for females (Tian et al., 2017), respectively. It is noteworthy that a recent meta-analysis and review of Chinese community-dwelling elderly individuals (aged 65 years and older) reported a higher prevalence rate of 17.4% compared to the earlier mentioned studies on Chinese community-dwelling older adults (Ren et al., 2022). One potential explanation for this difference could be the age range of individuals included (aged 65 years and older versus aged 60 years and older in the earlier studies).

These studies underscore the disease burden from sarcopenia among the older population globally, including in China. Additionally, sarcopenia is associated with an increased risk of developing a range of adverse health issues, such as functional decline, falls, fractures, and mortality (Cruz-Jentoft and Sayer, 2019). A recent nationwide longitudinal study in China has shown a connection between sarcopenia and a higher trajectory of activities of daily living disability in middle-aged and older adults (Lan et al., 2024). However, to date, the precise mechanisms underlying its pathogenesis are not fully elucidated. Therefore, a better understanding of the pathogenetic mechanisms is important to guide the development of therapeutic and interventional approaches.

### Advancements in the understanding of gut dysbiosis in primary sarcopenia

#### Gut–muscle axis: intricate interplay between the gut microbiota and skeletal muscle in sarcopenia

The connection of the gut microbiota with human health and diseases, including the gut–muscle axis and its role in sarcopenia, has attracted growing interest. The gut–muscle axis represents the bidirectional intricate crosstalk between the gut microbiota and skeletal muscles. Both the gut and skeletal muscles are recognized as having a signaling function, with skeletal muscle recently being shown to produce and release various myokines that have the potential to interact with distant organs or tissues (Hawley, 2020; Severinsen and Pedersen, 2020).

Skeletal muscle tissue is recognized as a protein reservoir in the human body, with muscle cells densely packed with contractile proteins in the cytoplasm (Sartori et al., 2021). This structural characteristic implies the critical impact of protein synthesis and degradation, which constantly turnover, on muscle mass and function. Specifically, alterations in muscle myofibrillar proteins directly influence changes in muscle mass. Maintaining the optimal balance between protein synthesis and breakdown is essential for muscle homeostasis. An excess of protein synthesis over breakdown may lead to muscle hypertrophy, contributing to increased muscle mass and improved muscle function (Sartori et al., 2021). Conversely, an imbalance favoring protein breakdown over synthesis may result in muscle atrophy, contributing to a loss of muscle mass and a decline in muscle function (Sartori et al., 2021). The balance between the synthesis and degradation of skeletal muscle proteins is vital for the mass and function of skeletal muscle, and multiple complex networks are involved (Schiaffino et al., 2013; Sartori et al., 2021). Several factors, such as exercise, nutrition, inflammation, and hormones, are well documented to impact this balance, while the gut microbiota and derived metabolites also have been demonstrated to affect the molecular pathways related to the skeletal muscle protein balance (Lahiri et al., 2019; Mancin et al., 2023).

A previous animal study using mice raised in a germ-free environment without gut microbiota showed muscle atrophy and the reduced expression of genes associated with skeletal muscle growth (Lahiri et al., 2019). In contrast, the transplantation of fecal material from pathogen-free mice to germ-free mice resulted in an increase in skeletal muscle mass and a decrease in markers of muscle atrophy (Lahiri et al., 2019). To gain insights into the signaling pathways involved in muscle atrophy in mice without gut microbiota, key genes associated with muscle atrophy were examined. The results indicated an increase in the expression of Atrogin-1 and Murf-1, which encode E3 ubiquitin ligases. These genes are known to be regulated by Forkhead box O (FoxO) transcription factors, and mice without gut microbiota exhibited elevated expression of FoxO3 (Sandri et al., 2004; Lahiri et al., 2019). Additionally, an increase in the phosphorylation of the catalytic domain of adenosine 5′-monophosphate-activated protein kinase (AMPK), which plays a critical role in controlling muscle fiber size by activating the FoxO-mediated protein degradation pathway, was observed (Greer et al., 2007; Lahiri et al., 2019). These studies suggest that the activation of the AMPK–FoxO3–Atrogin cascade could serve as a potential signaling pathway that partially elucidates muscle atrophy in mice without gut microbiota. Consistent with the findings from Lahiri’s study on mice lacking gut microbiota, research in humans has demonstrated the connection between muscle atrophy and a notable change in gut microbiota, specifically in the order Clostridiales and the family *Lachnospiraceae* (Jollet et al., 2021). Importantly, the family *Lachnospiraceae* has the capability to produce SCFAs and metabolize primary bile acids, which are initially synthesized in the liver, into secondary bile acids in the intestine (Vacca et al., 2020). While new insights that coordinate the gut–muscle axis and its involvement in maintaining skeletal muscle mass and function as well as the development of sarcopenia continue to evolve, one key mechanism through which the gut microbiota influence skeletal muscle is through the production of metabolites. Through these related metabolites and pathways, the compositional change of the gut microbiota can potentially lead to skeletal muscle atrophy (Mancin et al., 2023).

#### Association between gut microbiota and sarcopenia

The intricate relationship between the gut microbiota and skeletal muscle underscores the impact of its compositional disruption, known as gut dysbiosis, on muscle health and disorders, including sarcopenia in the elderly. Extensive studies in experimental animals have provided evidence supporting the close relationship between the gut microbiota and characteristic changes in muscle mass and function associated with sarcopenia (Liu et al., 2021). Wu et al. (2020) observed that Ghrelin-null (Ghrl −/−) mice exhibited microbial dysbiosis during their youth and decreased muscle mass in old age. In another study, decreased skeletal muscle mass and function were found to be linked to an altered composition of gut microbiota in mice (Lahiri et al., 2019). Additionally, the same study demonstrated that the depletion of gut microbiota in germ-free mice directly triggered muscle atrophy (Lahiri et al., 2019). Conversely, *Bacteroides fragilis* gnotobiotic mice showed a significantly enhanced muscle mass and function in comparison with germ-free mice (Lahiri et al., 2019). Various animal studies have utilized microbiome-transplanted models to explore the direct correlation between the gut microbiota and muscle, and most of the findings indicated a similar grip strength and muscle mass in recipient mice when compared to their donors, although certain studies have reported inconsistent results with no discernible differences in muscle function or mass following microbiome transplantation (Liu et al., 2021).

Human studies also have examined the link between the gut microbiota and characteristic changes associated with sarcopenia like muscle mass/function by comparing elderly individuals with and without sarcopenia. Observational studies have revealed a strong correlation between variations in the relative abundance of the gut microbiome and age-related muscle loss as well as diminished physical performance (Picca et al., 2019; Castro-Mejía et al., 2020; Ghosh et al., 2022). For example, Picca et al. conducted a study comparing the gut microbiota and inflammation levels as well as physical frailty in older individuals with and without sarcopenia (Picca et al., 2019). The study revealed altered gut microbiota compositions, with a decreased abundance of *Barnesiellaceae* and *Christensenellaceae* and an increased abundance of *Oscillospira* and *Ruminococcus* microbial taxa in older individuals with sarcopenia and physical frailty compared to those without these conditions (Picca et al., 2019). Moreover, older individuals with physical frailty and sarcopenia exhibited higher systemic inflammation levels compared to those without these conditions (Picca et al., 2019). Furthermore, the individuals with a higher muscle mass displayed a greater gut microbiota diversity (Castro-Mejía et al., 2020).

#### Causal link of gut dysbiosis to sarcopenia-related traits as recently revealed by Mendelian randomization

As discussed earlier in this review, previous studies have noted a connection between gut microbiota and sarcopenia, particularly in the elderly population. However, a definitive causal relationship, its mediators, and the underlying mechanisms have yet to be fully elucidated. In a recent study by Zhao et al., Mendelian randomization analysis, a widely used method to establish causal relationships between risk factors and outcomes, was conducted to investigate the potential causal association between gut microbiota and sarcopenia-related traits, such as a low HGS and appendicular lean mass (Zhao et al., 2023). The study, utilizing the Mendelian randomization approach, identified significant compositional changes in the gut microbiota at the family and genus levels (Zhao et al., 2023). Specifically, *Alcaligenaceae* at the family level (a group of Gram-negative bacteria within the class Betaproteobacteria, encompassing genera like *Alcaligenes*, *Bordetella*, and *Achromobacter*), Family XIII at the genus level (specifically the genus Family XIII AD3011 group), and *Paraprevotella* at the genus level (a bacterial genus commonly present in the human gut microbiota within the family *Lachnospiraceae*, characterized by anaerobic, Gram-positive, and nonspore-forming properties) were found to be significantly positively associated with the risk of a low HGS (all *p* < 0.05); in contrast, *Streptococcaceae* at the family level (a group of bacteria belonging to the phylum Firmicutes) showed a negative association with a low HGS (*p* < 0.05; Zhao et al., 2023).

Moreover, eight bacterial taxa (*Actinomycetaceae*, *Actinomycetales*, *Bacteroides*, *Bacteroidaceae*, *Marvinbryantia*, *Phascolarctobacterium*, *Prevotellaceae*, and *Porphyromonadaceae*) were linked to an increased risk of appendicular lean mass (*p* < 0.05), while the *Eubacterium fissicatena* group exhibited a negative association with the appendicular lean mass (*p <* 0.05; Zhao et al., 2023). These findings from the Mendelian randomization analysis suggest a causal link between specific compositional changes in the gut microbiota at the family and genus levels and traits related to sarcopenia. Furthermore, eight bacterial taxa (*Actinomycetaceae*, *Actinomycetales*, *Bacteroides*, *Bacteroidaceae*, *Marvinbryantia*, *Phascolarctobacterium*, *Prevotellaceae*, and *Porphyromonadaceae*) were significantly linked to a higher risk of appendicular lean mass (*p* < 0.05), with the *Eubacterium fissicatena* group displaying a negative association with the appendicular lean mass (*p* < 0.05; Zhao et al., 2023). These results from the Mendelian randomization analysis suggest a causal association of gut microbiota compositional changes at the family and genus levels with sarcopenia-related traits (Zhao et al., 2023), while factors that mediate the causal relation remain unexplored. Therefore, understanding how gut dysbiosis contributes to primary sarcopenia is crucial for developing new interventions.

#### The mechanisms that underlie the role of gut microbiota in affecting muscle mass and function in the development of sarcopenia

##### Gut microbiota–bile acids–FXR signaling pathway

Primary bile acids are initially produced in the liver and are secreted into the small intestine, where they undergo further metabolism into secondary bile acids by the gut microbiota, including microbiota-derived bile acids (Monte et al., 2009; Wahlström et al., 2016). The metabolism of bile acids in the intestine is predominantly regulated by the intestinal microbiota (Wahlström et al., 2016). Through the modulation of bile acid metabolism, at least in part, the gut microbial community plays a critical role in influencing systemic host metabolic processes, including glucose and lipid metabolism, systemic inflammation, as well as others associated with skeletal muscle. Recently, several studies have investigated the relationships between gut microbiota, bile acids, and FXR in association with skeletal muscle metabolism in mice (Qiu et al., 2021; Qiu et al., 2022), highlighting an increasing interest in the gut–muscle axis. Preclinical animal studies have revealed the impact of gut microbiota-derived bile acids and FXR signaling on skeletal muscle metabolism, leading to an enhancement in skeletal muscle growth and attenuation in skeletal muscle wasting (Qiu et al., 2021; Qiu et al., 2022). These seminal studies suggest a crucial role of the gut microbiota–bile acids–FXR signaling pathway in the gut–skeletal muscle axis, which may underlie the adverse effects of gut dysbiosis on muscle growth, mass, and function in the occurrence of sarcopenia in aged mice. However, the differential signaling properties of bile acids between humans and mice may impede the direct translation of findings from preclinical animal studies to human studies (Wahlström et al., 2016).

**Figure 1:**
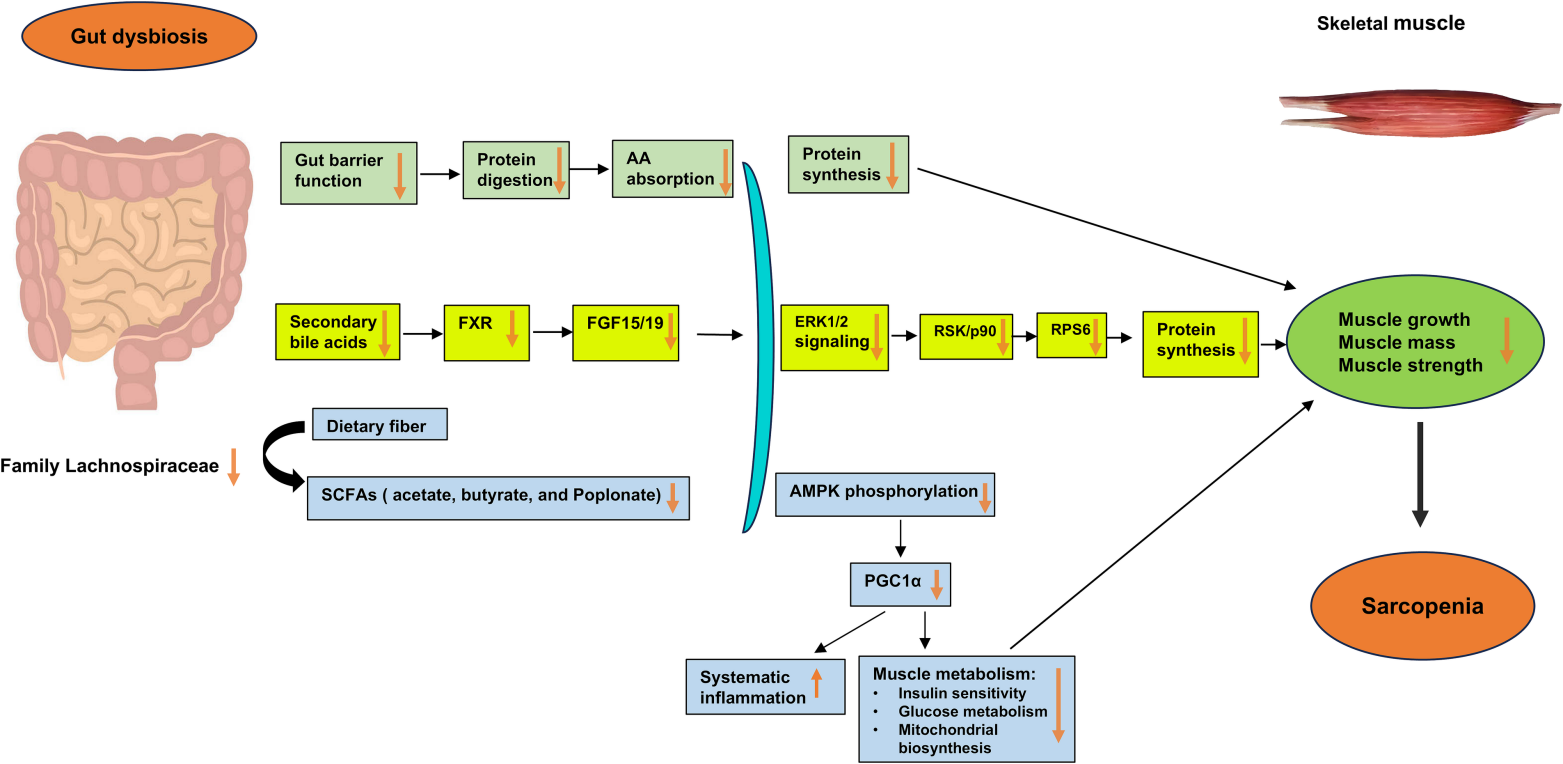
Potential mechanisms underlying the impact of gut dysbiosis on skeletal muscle mass and function in the development of sarcopenia. Gut dysbiosis, characterized by an imbalance in the gut microbiota, disrupts the gut barrier and impairs the production of microbiota-derived bile acids and short-chain fatty acids (SCFAs). In gut dysbiosis, the decrease or absence of bile salt hydrolase (BSH)-harboring bacteria diminishes the production of secondary deconjugated bile acids, leading to the accumulation of the primary bile acid tauro-*β*-muricholic acid (TβMCA). TβMCA is known to act as an antagonist of the farnesoid X receptor (FXR), and its increase during gut dysbiosis inhibits the FXR–fibroblast growth factor (FGF) 15/19 signaling pathway. This inhibition results in reduced phosphorylation of extracellular signal-regulated protein kinase (ERK) 1/2 and its downstream target p90 ribosomal S6 kinases (RSK/P90), along with decreased activity of ribosomal protein S6 (RPS6). Consequently, the downregulation of ERK signaling cascades impairs the muscle synthetic response due to decreased levels of FGF15/19, leading to a decrease in skeletal muscle mass and muscle atrophy. Through these major gut microbiota-derived metabolites and related signaling pathways, such as the bile acid–FXR–FGF15/19 signaling pathways, gut dysbiosis may lead to decreased muscle growth, mass, and strength, thereby contributing to the development of sarcopenia. AA, amino acid; AMPK, adenosine monophosphate-activated protein kinase; BSH, bile salt hydrolase; ERK, extracellular signal-regulated protein kinase; FGF, fibroblast growth factor; FXR, farnesoid X receptor; RPS6, ribosomal protein S6; RSK/p90, p90 ribosomal S6 kinases; SCFAs, short-chain fatty acids.

Based on existing studies, we have proposed the potential mechanisms underlying the development of sarcopenia through the gut dysbiosis–bile acids–FXR signaling pathway (Figure 1). In cases of gut dysbiosis, the decrease or absence of bile salt hydrolase (BSH)-harboring bacteria inhibits the production of secondary deconjugated bile acids, leading to an accumulation of the primary bile acid tauro-*β*-muricholic acid (TβMCA). TβMCA acts as an antagonist of FXR, and its increased levels during gut dysbiosis suppress the FXR–fibroblast growth factor (FGF) 15/19 signaling pathway, resulting in reduced phosphorylation of extracellular signal-regulated protein kinase (ERK) 1/2 and its downstream target p90 ribosomal S6 kinases (RSK/P90), along with decreased activity of ribosomal protein S6 (RPS6) (Benoit et al., 2017; Qiu et al., 2021; Qiu et al., 2022). Consequently, the downregulation of ERK signaling pathways impairs muscle synthetic response, leading to a decrease in skeletal muscle mass and muscle atrophy. The bile acid–FXR–FGF15/19 signaling pathways in gut dysbiosis may contribute to diminished muscle growth, mass, and strength, potentially culminating in the development of sarcopenia.

##### Gut microbiota-derived SCFAs potentially influencing skeletal muscle metabolism, mass, and function

SCFAs are bioactive compounds produced by gut bacteria through the fermentation of nondigestible dietary fiber (Krautkramer et al., 2021). Emerging evidence suggests that when SCFAs are absorbed from the gut lumen and enter either the circulation or the liver via the portal vein, they can act as second messengers for signal transduction and can regulate host metabolism in various tissues, thereby exerting a role in health and disease (Frampton et al., 2020; Li et al., 2021; Tan et al., 2021; Wang et al., 2024). This includes their potential role as a modulator of skeletal muscle metabolism, influencing its mass and function (Frampton et al., 2020). The three major SCFAs (acetate, propionate, and butyrate) have been studied for their impact on skeletal muscle, demonstrating an enhanced muscle condition (Lahiri et al., 2019). Several studies in humans have revealed alterations in the composition of gut microbiota and the production of SCFAs in response to exercise (Allen et al., 2018; Barton et al., 2018). Specifically, a decrease in the abundance of *Bacteroidetes* and an increase in fecal SCFA concentrations were observed in lean sedentary individuals who underwent a 6-week exercise training program (Allen et al., 2018). These changes in *Bacteroidetes* and fecal SCFA concentrations were abrogated when these participants returned to a sedentary lifestyle (Allen et al., 2018). At present, the impact of SCFAs on strength and endurance requires further investigation, but current evidence suggests that SCFAs derived from the gut microbiota may serve as a significant metabolic fuel for skeletal muscle (Frampton et al., 2020).

A recent human study examined the potential link between the gut microbiota composition and muscle atrophy by utilizing a severe hypoactivity model of dry immersion (Jollet et al., 2021). The resulting data suggest that severe physical inactivity-induced muscle atrophy is associated with an alteration in gut microbiota, including the family *Lachnospiraceae* and the order Clostridiales (Jollet et al., 2021). Notably, the family *Lachnospiraceae* has the capacity to produce SCFAs and microbiota-derived bile acids (Vacca et al., 2020). This study suggests that the family *Lachnospiraceae* can play a pivotal role in the hypoactivity–gut microbiota axis (Jollet et al., 2021). Supporting these observations, preclinical studies also have provided evidence that the presence of gut microbiota and related metabolites (e.g., SCFAs and bile acids) may be involved in the regulation of skeletal muscle metabolism (Mancin et al., 2023). Existing studies also have suggested that SCFAs (e.g., acetate, propionate, and butyrate) play a role in mediating the connection between the gut microbiota and skeletal muscle, thus affecting the mass and function of skeletal muscle through both direct and indirect effects (Frampton et al., 2020). The mechanisms governing the effects of gut microbiota-derived SCFAs on signaling pathways in skeletal muscle have been explored, revealing that SCFAs promote the phosphorylation of adenosine monophosphate (AMP)-activated protein kinase (AMPK) and potentially peroxisome proliferator-activated receptor (PPAR) gamma coactivator 1-alpha, through which SCFAs influence glucose and lipid metabolism in skeletal muscle (Frampton et al., 2020). Further investigations have shown that SCFAs can activate AMPK by increasing AMP concentrations, binding to G protein-coupled receptor 41 (GPR41) or GPR43, or activating PPAR delta (Frampton et al., 2020).

Despite complex influencing factors, SCFA concentrations typically decrease with age (Tan et al., 2014). Based on existing studies, it is proposed that aging induces dysbiosis of the gut microbiota (i.e., decreases in the family Lachnospiraceae), leading to a reduction in SCFA levels; in addition, age-related reductions in extracellular SCFA concentrations may adversely impact skeletal muscle through its receptors for SCFAs, specifically free fatty acid receptor (FFAR)2 and FFAR3 (Tan et al., 2014; Baxter et al., 2019; Huang W. et al., 2020; Vacca et al., 2020; Tang et al., 2022). Among these receptors, FFAR2 is considered the primary receptor for SCFAs, and all SCFAs (acetate, propionate, and butyrate) can stimulate FFAR2 in mice (Tang et al., 2022). Notably, mice treated with the SCFA butyrate exhibited a significant improvement in the muscle mass of the hindlimb and gastrocnemius by ameliorating muscle atrophy through the stimulation and upregulation of FFAR2 (Tang et al., 2022). Mechanistic insight further revealed that the beneficial effects of SCFAs on muscle atrophy were achieved by the FFAR2-mediated activation of the PI3K/Akt/mTOR pathway (Tang et al., 2022). However, the precise molecular mechanisms underlying the relationship between age-related reductions in SCFA concentrations and muscle loss in primary sarcopenia remain largely unknown and require further research. Understanding the mechanistic links between SCFAs and skeletal muscle in the development of primary sarcopenia can provide insights into potential strategies to mitigate the effects of declining SCFA levels on muscle health in aging individuals and thereby prevent primary sarcopenia.

Collectively, the emerging evidence suggests a significant role of gut microbiota in regulating skeletal muscle metabolism and function, potentially mediated by its derived SCFAs and the conversion of hepatic bile acids in the gut. Building upon the aforementioned studies, we propose potential mechanisms underlying the impact of gut dysbiosis on skeletal muscle mass and function in the development of sarcopenia (Figure 1). However, further in-depth research is required to understand the precise metabolic mechanisms involving gut microbiota-derived SCFAs and bile acids, systemic metabolism, systemic inflammation, and skeletal muscle metabolism concerning skeletal muscle mass, function, and related conditions such as primary sarcopenia. Moreover, future research should prioritize investigating the effects of gut microbiota-derived SCFAs and bile acids on skeletal muscle in human subjects to validate the predominantly animal and cell culture findings and proposed mechanisms as well as to facilitate the translation of these discoveries into potential treatments or interventions.

### Implications for potential diagnosis and interventions for primary sarcopenia, challenges, and future directions

#### Implications for potential diagnosis

The significant alterations in gut microbiota associated with sarcopenia make them potentially valuable as noninvasive markers for this skeletal muscle disorder. Currently, research on the development of gut microbiota-based markers/predictive models for sarcopenia in the elderly is in its infancy. Nevertheless, recent years have witnessed advancements in this research area (Wang et al., 2022; He et al., 2023; Zhou et al., 2023; Guo et al., 2024). Accordingly, we have compiled a summary of potential gut microbiota-based biomarkers/predictive models and their performance for sarcopenia in Table 1. While the gut microbiota-based predictive models presented in Table 1 have achieved relatively high area under the receiver operating characteristic curve (AUC) values, indicating a good ability to discriminate between sarcopenia and nonsarcopenia, especially in population-based studies as reported by Wang et al. (2022) with an AUC of 0.852 in a predictive model based on differentially abundant gut microbiota species between sarcopenia and nonsarcopenia individuals (Wang et al., 2022), there are notable limitations. These limitations include a relatively small sample size and the lack of validation with external samples, among others. Further studies are necessary before translating these preliminary findings into clinical applications.

**Table 1:** Summary of potential gut microbiota-based biomarkers/predictive models and their performance for sarcopenia.

Study (Authors, year)	Study subjects and groups	Main gut microbiota alterations	Performance metrics of gut microbiota-based markers/ models for sarcopenia
Wang et al. (2022)	Individuals with sarcopenia (mean age, 72.2 years; *n* = 141) and nonsarcopenia (mean age, 62.3 years; *n* = 1,276)	Differentially abundant species in individuals with sarcopenia vs. nonsarcopenia individuals	Random forest model: AUC, 0.852
Zhou et al. (2023)	Patients with sarcopenia (age > 60 years; *n* = 30) and patients without sarcopenia (age > 60 years; *n* = 30)	*Blautia* (genus-level biomarker); *Lachnospiraceae*_unclassified (genus-level biomarker); *Subdoligranulum* (genus-level biomarker) in sarcopenia vs. nonsarcopenia	AUC, 0.7944 AUC, 0.7889 AUC, 0.7633
He et al. (2023)	Patients with sarcopenia (age, 62–88 years; *n* = 32) and healthy older adults as controls (age, 60–79 years; *n* = 31)	20 bacterial species-level biomarkers of gut microbiota in sarcopenia patients vs. healthy controls; 34 gut microbiota-related metabolites in sarcopenia patients vs. healthy controls	Supervised classification model: AUC, 0.7083–0.8833 Random forest algorithm: AUC, 0.9223–0.9833
Guo et al. (2024)	Older adults with low HGS (age, 77–90 years; *n* = 8) and matched individuals with normal HGS (age, 77–90 years; *n* = 7)	Increased abundance of the genera *Parabacteroides* in low HGS vs. normal HGS; Increased abundance of the genera *Intestinibacter* in low HGS vs. normal HGS	*p*-value, 0.006536; FDR, 0.466388 *p*-value, 0.008969; FDR, 0.466388

Sarcopenia was diagnosed according to the Asian Working Group for Sarcopenia (AWGS) in 2019 criteria. AUC, area under the receiver operating characteristic curve; FDR, false discovery rate; HGS, handgrip strength.

#### Implications for the development of novel interventions for primary sarcopenia

The findings on the role of gut microbiota in the metabolism of skeletal muscle, muscle mass, and strength/function, along with the underlying mechanisms, have significant implications for managing primary sarcopenia. Direct microbiota-related interventions, such as probiotic supplementation and fecal transplantation, whether used alone or in combination with nonpharmacological approaches, have been investigated. Probiotics, which are nonpathogenic living organisms, are well known for their beneficial effects on host health and disease prevention, leading to a consensus statement on the appropriate use of probiotics (Hill et al., 2014). Recent research has explored various nutritional strategies, including probiotic supplementation (e.g., *Bacillus coagulans* GBI-30 and *Bacillus subtilis*), to enhance muscle mass and function (Jäger et al., 2016; Toohey et al., 2020). In addition, studies have demonstrated that probiotic supplementation can effectively enhance muscle mass and function in experimental animal models (Giron et al., 2022). In the context of age-related sarcopenia, specific strains of probiotics, such as lactobacilli and bifidobacteria, have shown promise in mitigating age-related muscle loss in various mouse models (Ni et al., 2019; Chen et al., 2022). Several animal studies in mice or rats also have investigated the potential benefits of probiotic supplements, either alone or in conjunction with exercise, on muscle mass, growth, or function (Chen et al., 2016; Chen et al., 2019; Ni et al., 2019; Soares et al., 2019; Huang W. C. et al., 2020; Lee et al., 2020; Chen et al., 2022). The probiotic supplements examined include *Lactobacillus plantarum* TWK10 (LP10) (Chen et al., 2016), *Bifidobacterium longum* BL986 and *Lactobacillus casei* LC122 (Ni et al., 2019), *Lactobacillus paracasei* PS23 (Chen et al., 2019), *Saccharomyces boulardii* (Soares et al., 2019), *Lactobacillus salivarius* SA-03 (Lee et al., 2020), and *Bifidobacterium longum* OLP-01 (Huang W. C. et al., 2020). Moreover, the beneficial effects of probiotic supplements on muscle growth were demonstrated by Ni et al. (2019) and Chen et al. (2016), and Chen et al. (2019), while their impact on muscle function was shown by Ni et al. (2019), Soares et al. (2019), Chen et al. (2016), Chen et al. (2019), Lee et al. (2020), and Huang W. C. et al. (2020). Given the promising results from animal studies, it would be worthwhile to assess the beneficial effects of these probiotic supplements, either alone or in combination with exercise and anti-inflammatory agents, in elderly individuals with sarcopenia.

SCFA-producing gut microbes are influenced by various substances in foods and supplements (Cronin et al., 2021; Fusco et al., 2023). Specifically, non-starch polysaccharides, resistant starch, and resistant oligosaccharides in high-fiber diets, as well as supplementary fiber, inulin, and fructooligosaccharides in prebiotic supplements, have been shown to directly promote these gut microbiomes, leading to increased SCFA production (Cronin et al., 2021; Fusco et al., 2023). These findings suggest that maintaining a well-balanced diet rich in fiber and prebiotics is generally beneficial for supporting SCFA-producing gut microbes and enhancing SCFA production, potentially exerting beneficial effects on sarcopenia.

However, translating these findings from animal studies to human applications poses challenges due to the limited number of studies and the complexities involved in accurately and consistently assessing muscle mass and function in older individuals (Liu et al., 2021). Currently, several clinical trials have evaluated the effects of prebiotics, probiotics, or symbiotics on sarcopenia parameters in the elderly (Neto et al., 2013; Buigues et al., 2016; Huang et al., 2019). For example, Buigues et al. conducted a randomized placebo-controlled trial, demonstrating that supplementation with a prebiotic formulation (Darmocare Pre®), comprising a combination of inulin and fructooligosaccharides (targeting microbiota), improved two aspects of frailty syndrome—time to exhaustion and grip strength—in older participants (aged 65 years and above) (Buigues et al., 2016). Similarly, young healthy adults consuming *Lactobacillus plantarum* TWK10 daily experienced a dose-dependent enhancement in antifatigue capacity as well as an increased muscle mass (Huang et al., 2019). These two clinical trials consistently supported the beneficial effects of probiotics and prebiotics on muscle mass and strength (Buigues et al., 2016; Huang et al., 2019). In contrast, a small-scale clinical study involving only 17 participants found that the long-term use of a synbiotic (consisting of fructooligosaccharides, *Bifidobacterium lactis*, and *Lactobacillus* strains) did not enhance the grip strength or fat-free mass in the elderly (Neto et al., 2013). Recent randomized controlled trials investigating the beneficial effects of probiotic supplements on sarcopenia parameters and a meta-analysis of seven studies are summarized in Table 2 (Ford et al., 2020; Karim et al., 2022a; Karim et al., 2022b; Nistor-Cseppento et al., 2022; Rondanelli et al., 2022; Prokopidis et al., 2023; Shokri-Mashhadi et al., 2023; Qaisar et al., 2024).

**Table 2:** Summary of recent human studies on the beneficial effects of probiotic supplements on sarcopenia parameters.

Study	Design/Intervention	Study subjects	Sample size	Groups	Key findings (Measurements for sarcopenia)
Ford et al. (2020)	RCT/18-week HPD plus probiotic	Elderly women (mean age, 73.7 years)	26	HPD+ multistrain probiotic	Muscle mass: increased
Karim et al. (2022a)	RCT/16-week Vivomix (112 billion)	COPD patients (male, aged 63–73 years)	100	Probiotic vs. placebo	HGS: improved SPPB scores: increased Gait speed: improved
Karim et al. (2022b)	RCT/12-week multistrain probiotic	CHF patients	92	Probiotic vs. placebo	HGS: improved SPPB scores: increased Gait speed: improved
Rondanelli et al. (2022)	RCT/2-month n-3FAs plus Leucine plus probiotic (LPPS23)	Patients with sarcopenia^#^ (mean age, 79.7 years)	50	n-3FAs plus Leucine plus probiotic vs. placebo	ALM: increased HGS: improved SPPB scores: increased
NIstor-Cseppento et al. (2022)	RCT/2-month high-protein +probiotics	Sarcopenia patients with COVID-19	200	High-protein plus probiotics vs. placebo	Skeletal muscle index: increased
Prokopidis et al. (2023)	Meta-analysis/≥12-week probiotic	Adults (age > 18 years) from RCTs	NA	Probiotic vs. placebo	Muscle mass: improved Global muscle strength: increased
Shokri-Mashhadi et al. (2023)	Meta-analysis/>12-week probiotic	Participants from 7 studies	701	Probiotic vs. placebo	Muscle mass: increased Muscle strength: increased Muscle function: improved
Qaisar et al. (2024)	RCT/16-week Vivomix (112 billion)	Geriatric men with sarcopenia	123	Probiotic vs. placebo	Sarcopenia-related QoL scores: increased HGS: increased

ALM, appendicular lean mass; CHF, chronic heart failure; COPD, chronic obstructive pulmonary disease; COVID-19, coronavirus disease 2019; HGS, handgrip strength; HPD, high-protein diet; NA, not available; QoL, quality-of-life; LPPS23, *Lactobacillus paracasei* PS23; n-3FAs, omega-3 fatty acids; RCT, randomized controlled trial; SPPB, short physical performance battery. ^#^Sarcopenia was diagnosed based on the revised European Consensus on Definition and Diagnosis criteria.

Analysis of the randomized controlled trials revealed that probiotic supplementation enhanced muscle strength (handgrip strength) and physical performance and function (gait speed), indicating a beneficial impact on overall sarcopenia parameters in older adults (Prokopidis et al., 2023; Besora-Moreno et al., 2024). While the evidence for the efficacy of prebiotics remains scarce, a randomized clinical trial demonstrated a significant enhancement of handgrip strength, a key parameter of sarcopenia, in frail older individuals aged 65 and above, following the consumption of a prebiotic product consisting of inulin and fructooligosaccharides compared to a placebo (Buigues et al., 2016). Additionally, the beneficial effects of a certain probiotic supplement, Bifidobacteria, on sarcopenia parameters have been observed in Asian older adults but not in European populations, suggesting a potential specificity of these effects (Prokopidis et al., 2023). Therefore, further randomized clinical trials are needed to assess the effects of prebiotics, identify the more efficacious probiotic strains, and explore the potential synergistic effects of combining prebiotics and probiotics for better management of sarcopenia in older adults.

#### Challenges and future directions in primary sarcopenia research

Despite the rapid progress in understanding the role of gut microbiota in primary sarcopenia, potentially through their metabolites, and observing the beneficial effects of probiotics, either alone or in combination with nonpharmacological approaches, on improving sarcopenia indices in animal and human studies, there are limitations and challenges that need to be addressed by future studies. (1) Standardized diagnostic criteria for primary sarcopenia have not been universally agreed upon. Various international groups, such as IWGS, AWGS, EWGSOP, and FNIH, have each established their own diagnostic criteria and definitions for sarcopenia. These varying criteria may impact both research and treatment strategies for sarcopenia. In research, heterogeneous criteria are associated with different outcomes, affecting study consistency and comparability (Han et al., 2018). Treatment strategies are also influenced, as differences in diagnostic criteria may affect early identification, treatment initiation, and monitoring. Tailoring treatments based on specific criteria is crucial for optimal outcomes, including exercise interventions, and physical or nutritional therapy (Ooi and Welch, 2024; Han et al., 2018). Therefore, the absence of a universally uniform definition and diagnostic criteria for sarcopenia will require standardization. (2) Sarcopenia is more prevalent in males than in females (Cruz-Jentoft and Sayer, 2019). Given the sex-specific differences in the gut microbiota composition (Kim et al., 2020), future research should explore the impact of sex hormones and their interplay with the gut microbiota. These sex-specific differences in the gut microbiome might contribute to the sex dimorphism in sarcopenia. In addition, further investigations might help to elucidate the intricate pathological mechanisms underlying the sex disparity in primary sarcopenia, particularly the higher prevalence in males compared to females. Moreover, it would also be worthwhile to explore the potential role of hormone therapy in the elderly for the prevention or treatment of sarcopenia in the elderly. (3) In a recent study by Zhao et al., a Mendelian randomization analysis was conducted to investigate the potential causal association between the gut microbiota and sarcopenia-related traits, including appendicular lean mass and low HGS (Zhao et al., 2023), indicating the definitive causal relationship between the gut microbiota and sarcopenia. While this study indicated a definitive causal relationship between the gut microbiota and sarcopenia, the mediators and underlying mechanisms remain incompletely elucidated, necessitating further research in future studies. (4) The existing evidence suggests that key metabolites of the gut microbiota, including SCFAs and secondary bile acids, can mediate their effects on the metabolism of skeletal muscle and, consequently, the indices of sarcopenia. For example, the beneficial effects of SCFAs on muscle mass and function have been demonstrated in animal models of sarcopenia. Clinical studies also have shown associations between the gut microbiota composition, SCFA levels, and sarcopenia in elderly individuals. However, further investigation is needed to establish the direct causal relationship between gut microbiota-derived SCFAs and the development of primary sarcopenia through well-designed longitudinal studies and intervention trials. Gut microbiota-derived SCFAs have emerged as potential novel therapeutic agents for enhancing muscle mass (Liu et al., 2021). Furthermore, future human studies are crucial for confirming the promising results from experimental cell culture or animal studies, as this validation is essential for facilitating the translation of these findings into potential treatments or interventions. (5) An integrated approach, combining probiotics with nonpharmacological strategies like high-protein intake, exercise, and an anti-inflammatory diet, merits exploration in future research. Dietary modifications, exercise, and fecal microbiota transplantation represent diverse methods to rebalance the gut microbiota and to alleviate the effects of dysbiosis on skeletal muscle. Tailored interventions should be guided by individual microbiota profiles to enhance treatment effectiveness for sarcopenia in the elderly. (6) A key focus for future research is to identify gut bacteria or their metabolites that are unique to sarcopenia in the elderly, with the potential to serve as biomarkers for distinguishing older individuals with or without sarcopenia.

## Conclusion

In conclusion, the role of the gut microbiota-muscle cross-talk in primary sarcopenia has been increasingly supported by multiple lines of evidence, including the association between gut dysbiosis and sarcopenia, as well as the causal link of gut dysbiosis to sarcopenia. Mechanistic studies, predominantly conducted in animals and cell cultures, have suggested that gut dysbiosis may play a causal role in primary sarcopenia through the modulation of key metabolites, including gut microbiota-derived SCFAs, secondary bile acids, and their associated signaling pathways. Specifically, the age-related decline in SCFA-producing gut microbiomes leads to a reduction in SCFAs, through an AMPK-mediated regulatory pathway that modulates PGC-1α gene expression, affecting skeletal muscle protein metabolism and potentially contributing to sarcopenia. Moreover, gut microbiota dysbiosis may disrupt the secondary bile acids-FXR signaling pathway and skeletal muscle protein synthesis, potentially resulting in the development of sarcopenia. These findings have important clinical implications by providing potential therapeutic targets for sarcopenia and identifying gut bacteria or metabolites specific to sarcopenia as potential biomarkers to differentiate older individuals with or without sarcopenia. Future research should prioritize assessing the impact of gut microbiota-derived SCFAs and bile acids on skeletal muscle in human subjects, validating the findings from animal and cell culture studies, and facilitating the translation of discoveries into practical treatment and diagnostic options. Furthermore, the exploration of direct microbiota-related interventions, such as probiotic supplementation and fecal transplantation, alongside nonpharmacological approaches like a high-protein diet and exercise require further large-scale clinical trials to offer additional evidence and ultimately improve care for primary sarcopenia.
